# The Effects of Socioeconomic Determinants on Hypertension in a Cardiometabolic At-Risk European Country

**DOI:** 10.1155/2017/7107385

**Published:** 2017-08-28

**Authors:** Sarah Cuschieri, Josanne Vassallo, Neville Calleja, Nikolai Pace, Julian Mamo

**Affiliations:** ^1^Department of Anatomy, Faculty of Medicine and Surgery, University of Malta, Msida, Malta; ^2^Faculty of Medicine and Surgery, University of Malta, Msida, Malta; ^3^Department of Public Health, Faculty of Medicine and Surgery, University of Malta, Msida, Malta; ^4^Department of Health Information and Research, Ministry of Health, Guardamangia, Malta

## Abstract

**Background:**

A relationship has been established between socioeconomic status and hypertension. The aim of this study was to determine the prevalence of hypertension and to explore the links between hypertension and socioeconomic factors in the adult population of Malta.

**Methods:**

A national representative cross-sectional health examination study was performed between 2014 and 2016. Sociodemographic and medical history data was gathered by validated questionnaires while blood pressure was measured. Prevalence rates of known hypertension, newly hypertension, and global hypertension were calculated. Associations between sociodemographic characteristics and hypertension were identified through logistic regression models.

**Results:**

Hypertension contributed to 30.12% (CI 95%: 28.71–31.57) of the study population, with a male preponderance. The majority was known hypertensive (73.59% CI 95%: 71.01–76.02), with only three-quarters on medication. Multivariant analyses showed that increasing age and body mass index, male gender, and living in Gozo, Western district, and Northern Harbour district were associated with having hypertension.

**Conclusion:**

Hypertension is a problem in Malta especially in the male population and with increasing age and body mass index. Education did not exhibit any associated risk for having hypertension, which is inconsistent with the literature, while habitat localities played a role in hypertension development.

## 1. Introduction

High blood pressure is a serious public health concern, with the World Health Organization (WHO) reporting a hypertension global mortality rate of 13% [1]. Hypertension is estimated to contribute to 25% of all European myocardial infarctions [2]. In fact, the WHO European Region document* Health 2020 Policy* gave hypertension high priority in order to tackle the epidemic and to reduce its prevalence [3]. High blood pressure is a preventable disease and has been associated directly with lifestyle habits, including tobacco smoking, a lack of physical activity, and alcohol consumption [2]. In fact, the relationship between hypertension and socioeconomic status has been well established [4].

Socioeconomic status (SES) is a complex term combining a number of variables, including employment status, educational level, income, and wealth as well as place of residence. SES is a well-established cardiovascular risk factor and means for predicting behaviour [5, 6]. The educational level has been established as the best marker of SES since it offers the most stable measure at an individual level and does not have the problem of reverse causation such as income and wealth status [6].

Malta is a Mediterranean island at crossroads between Europe and Africa, with 40.1% of the mortality rate attributed to cardiovascular disease [7]. Considering the small size of this island and its crucial location, a representative adult health examination cross-sectional study was considered feasible to provide much needed information on health effects. The aim of this study was to determine the prevalence of hypertension and to explore the links between hypertension and socioeconomic factors in the adult population of Malta.

## 2. Method

A cross-sectional study was conducted by the University of Malta between November 2014 and January 2016, with the survey name of “SAHHTEK”* (your health). *The detailed survey methodology was described elsewhere [8]. In summary, a randomized stratified sample, by age (18 to 70 years) and gender, representing approximately 1% of the total population from each Maltese town, was invited to participate in the health examination survey. A validated sociodemographic and medical history questionnaire was filled in during the survey [8]. The health examination consisted of three consecutive blood pressure readings after a 20-minute rest (sitting down), besides anthropometric measurements. The average of the three blood pressure readings was recoded and used for this study. Informed consent was obtained from every participant. Ethical and data protection approvals were granted from the University of Malta Research Ethical Committee (UREC) and the Information and Data Protection Commissioner, respectively.

The population reporting a history of hypertension or who was on antihypertensive medication was labeled as* “known hypertensive.”* Participants with a systolic blood pressure over 140 mmHg or a diastolic blood pressure above 90 mmHg and who were not* known hypertensives *were labeled as* “newly hypertensive.”* Those reporting to suffer from* known hypertension *but did not report to be on treatment and were found to have a normal blood pressure during health examination were labeled as* normotensive*. All population with no history of hypertension and a normal blood pressure during health examination was also labeled as* normotensive*.

The* known hypertensive *and the* newly hypertensive* subpopulations were considered collectively as the global* hypertension population* for this study. The prevalence rates of* known hypertension, newly hypertension, *and the global* hypertension population* were calculated. The study population was age-stratified by 10-year age groups (20 to 69 years), and considering the representative prevalence rates in these age groups, an estimate of the total Maltese adult population suffering from hypertension was calculated.

The links between self-reported sociodemographic, lifestyle variables (residing district, highest educational level, employment status, smoking habits, alcohol consumption habits, and physical activity levels), body mass index (BMI), and hypertension were explored. Univariate and multiple variant logistic regression models were performed to assess any associations between these sociodemographic characteristics and hypertension.

## 3. Results

**Figure 1:**
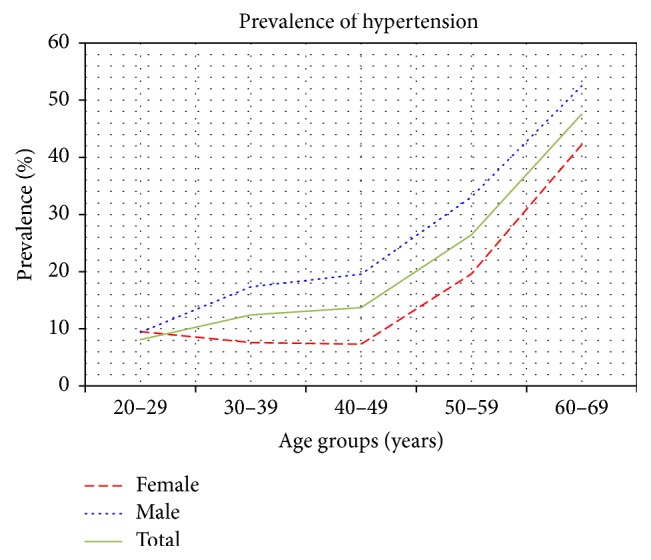
Distribution of hypertension prevalence by age groups and gender.

The survey response rate was of 49% (*n* = 3947), out of which 30.12% (CI 95%: 28.71–31.57) contributed to the hypertension population (*n* = 1189). Hypertensive males accounted for 35.04% (CI 95%: 32.97–37.15) of the total study male population, while hypertensive females accounted for 25.09% (CI 95%: 23.21–27.06) of the total female study population. On age and gender stratification, a male predominance was present throughout all age groups, as seen in Figure 1. With increasing age in the 20- to 39-year bracket, males had higher blood pressure levels, while the female population actually had slightly lowered prevalence with increasing age in this bracket. However, from the age of 40 years, a higher prevalence rate of hypertension was exhibited for both genders with every age increase. On applying the prevalence rates for each age group and gender to the total Maltese population by age and gender [9], an estimated distribution of hypertension within the Maltese population was established, as seen in Table 1.

The majority of the hypertension population consisted of* known hypertensives* (73.59% CI 95%: 71.01–76.02), with the male prevalence of 71.29% (CI 95%: 67.82–74.52) and a female prevalence of 76.89% (CI 95%: 72.95–80.42). Among the* known hypertensives*, 85.60% (CI 95%: 83.11–87.78) were on medication. However, 5.34% (CI 95%: 3.93–7.21) were found to have uncontrolled systolic blood pressure (>140 mmHg) while 1.20% (CI 95%: 0.60–2.30) had uncontrolled diastolic blood pressure (>90 mmHg) during the health examination survey. Out of those reporting to suffer from hypertension, 5.52% (CI 95%: 4.19–7.23) did not report to be on treatment and were found to have a normal blood pressure during the health examination. This subgroup may have exhibited an error in their medical history recall and were therefore considered to be normotensive.

The* newly hypertensive* population contributed to 26.41% (CI 95%: 23.98–28.99) of the hypertension population. The majority (64.01% CI 95%: 58.56–69.13) of the newly diagnosed hypertensives were male.  Table 2 demonstrates different hypertension characteristics by gender. The Maltese males tend to be more likely to have an elevated blood pressure, be unaware of their hypertension, be untreated, or have uncontrolled blood pressure.

## 4. Hypertension Population and Sociodemographic Characteristics

On comparing the sociodemographic characteristics and lifestyle factors to the hypertensive and normotensive populations, significant differences were observed. These differences are outlined in Table 3.

Multivariant analyses showed that increasing age and increasing BMI, the male gender, physical activity, and living in Gozo, Western district, and Northern Harbour were associated with the development of hypertension (Table 4).

Alcohol consumption, smoking habit, education level, and employment status were not found to have a significant associated risk on hypertension after adjustment for all sociodemographic and lifestyle habits.

## 5. Discussion

Hypertension is one of the leading causes of cardiovascular disease and mortality worldwide [10]. The prevalence of hypertension varies from one global region to another, with the highest prevalence being in the African region (46%) and the lowest in America (35%), with the European region prevalence rate reported to be somewhere in between these two continents [11, 12]. The male gender, in our study, exhibited the highest hypertension prevalence, which is consistent with what is normally found within high-income countries [13].

On comparing the hypertension prevalence found in our study to a Pan-European study conducted in 5 European countries, Malta exhibited the highest hypertension prevalence [14]. This Pan-European study had reported that Germany had the highest hypertension prevalence among UK, Italy, France, and Spain. Malta showed higher prevalence rates for each gender (Germany male: 25.1%, female: 22.9%; Malta male: 36.49%, female: 27.14%), although one needs to keep in mind that different age bands (Malta study: 18 to 70 years, Pan-European study: 20–79 years) were examined in both studies [14]. Conversely, our study population reported Maltese hypertensives having a higher antihypertensive pharmacological prescription frequency when compared to the average European prescription frequency [15, 16]. Nonadherence to antihypertensive medication is a global issue and leads to loss of clinical efficacy as well as to loss of economic efficiency resulting in an increase in hospitalization costs [16].

Hypertension determinants are well established and include age, gender, sociodemographic factors, obesity, smoking, excess alcohol, and sedentary lifestyle [17, 18]. On univariant analysis, education level, which is considered as the most stable determinant of socioeconomic status, was found to exhibit an associated protective effect against having hypertension in our study. This effect was lost on adjusting for age and gender, which could be the result of a change in education opportunities over time and more females are continuing their education. This finding is inconsistent with other association reports where the level of education was found to have an inverse association with blood pressure [19, 20].

The female gender in our study was found to exhibit an associated protective effect on hypertension. This is partially consistent with findings from other studies within other high-income countries [21]. However, this protective effect exhibited by our female population was previously reported in middle-income countries and not high-income countries like Malta [22–24].

Intense physical activity was found to increase the risk of having hypertension in our study, which is not consistent with the literature [25]. A possible reason to this finding follows the fact that the Maltese population is well known to be at high risk of metabolic diseases. A proportion of the population follows lifestyle interventions to try to halt the development of these noncommunicable diseases, which may have led to this finding.

A link was established between three different districts (Gozo, Western, and Northern Harbour) and having hypertension. Further research is required to assess whether an environmental or a genetic cause may be contributing to this relationship. However, a possible explanation for the link between the Northern Harbour and hypertension could result from the fact that the majority of the Maltese population lives in this district.

A link has been reported between alcohol consumption and blood pressure, with chronic drinkers having a longer impact on blood pressure outcomes [26]. A linear relationship had been reported between alcohol intake and blood pressure [27]. This was not the case in our study, where nonalcohol drinkers were found to be at risk of having hypertension. However, this associated effect of alcohol consumption on hypertension development was lost once various confounder factors were adjusted for, suggesting that no direct link between alcohol and hypertension was evident in our population.

## 6. Study Limitations and Strengths

The response rate for the health examination was rather low due to the invasive blood measurement required, which may have affected the results. The demographic data and lifestyle habits were self-reported by participants. This predisposes the risk of human bias or inaccurate recollection of information. Blood pressure measurement is very sensitive; accuracy may have been affected by the behaviour of the participant, the environment of the setup, and the aneroid sphygmomanometer device used even though sphygmomanometers were calibrated regularly. Auscultation fieldworker observer errors may have occurred including systematic error in auscultation method if the observer did not hear well enough or had slow reactions to auditory and visual cues, even though fieldworkers were trained and their readings double checked with other experienced blood pressure measurers prior to initiation of the fieldwork. Measurement of blood pressure was taken after 20 minutes of sitting down; this avoided any blood pressure fluctuations. All participants were asked to sit up straight with both feet touching the floor and their arms resting at the heart level, where these techniques were found to enable more accurate readings. The study was a cross-sectional study, which does not adjust for changes in behaviour after the diagnoses of hypertension or other diseases. There is thus great difficulty in linking exposures with outcomes from this study in any causative way.

## 7. Conclusion

Approximately a third of the adult population in Malta (mostly males) suffered from hypertension. Aging males, increase in BMI, and living in Gozo, Western district, and Northern Harbour exhibited a higher associated risk to develop hypertension. Education levels were not found to be associated with the hypertension development.

## Figures and Tables

**(a):** 

Total Maltese population by age^*∗*^		Total population with hypertension	Percentage with hypertension, by age
20–29	55,218	5,345	9.68%
30–39	56,184	7,193	12.80%
40–49	48,406	6,466	13.36%
50–59	57,291	15,223	26.57%
60–69	55,986	26,395	47.15%

*Total*	273,085	60,622	

**(b):** 

Total male Maltese population by age^*∗*^		Male with hypertension	Percentage with hypertension, by age
20–29	28,228	2,646	9.38%
30–39	28,837	5,005	17.36%
40–49	24,546	4,796	19.54%
50–59	28,822	9,529	33.06%
60–69	27,388	14,384	52.52%

*Total*	137,821	36,360	

**(c):** 

Total female Maltese population by age^*∗*^		Female with hypertension	Percentage with hypertension, by age
20–29	26,990	2,699	10.00%
30–39	27,347	2,188	8.00%
40–49	23,860	1,670	7.00%
50–59	28,469	5,694	20.00%
60–69	28,598	12,011	42.00%

*Total *	135,264	24,262	

^*∗*^Demographic data 2013.

**Table 2:** Demonstrating hypertension characteristics, by gender.

Hypertension characteristics	Male (%) [*n* = 1998]	Female (%) [*n* = 1949]	Total (%) [*n* = 3947]
Normotensive	1298 (64.96)	1460 (74.91)	2758 (69.88)
Hypertensive *(global hypertension)*	700 (35.04)	489 (25.09)	1189 (30.12)
Unaware *(newly hypertensive)*	201 (10.06)	113 (5.80)	314 (7.96)
Aware *(known hypertensive)*	499 (24.97)	376 (19.29)	875 (22.17)
Untreated	81 (4.05)	45 (2.31)	126 (3.19)
Treated	418 (20.92)	331 (16.98)	749 (18.98)
Uncontrolled	32 (1.60)	15 (0.77)	47 (1.19)
Controlled	386 (19.32)	316 (16.21)	702 (17.79)

Total with elevated blood pressure	668 (33.43)	474 (24.32)	1142 (28.93)

**Table 3:** Distribution of the sociodemographic characteristics in the normotensive and the hypertensive populations by gender.

		Hypertensive (total)	Normotensive	*p* value^*∗*^	Hypertensive		*p* value^*∗*^
					Male (*n* = 729)	Female (*n* = 529)	
Locality	Southern Harbour	585	215	**0.0001**	124	91	**0.0001**
	Northern Harbour	731	340		195	145	
	Southeastern	382	220		122	97	
	Western	360	186		109	77	
	Northern	423	163		91	72	
	Gozo	208	134		87	46	

Education	Low educational level	361	273	**0.0001**	160	201	**0.0001**
	Medium educational level	497	1034		284	213	
	High educational level	399	1382		285	114	

Employment status	Employed	625	1898	**0.0001**	473	153	**0.0001**
	Not in employment	632	791		256	376	

Smoking habit	Smoking	268	691	**0.003**	192	76	**0.001**
	Nonsmoking	990	1999		537	453	

Alcohol consumption	Alcohol consumption	1487	625	**0.001**	458	167	**0.0001**
	No-alcohol consumption	1203	633		271	361	

Physical activity	No activity	290	98	**0.015**	45	53	0.084
	Walk	522	230		138	91	
	Moderate activity	1585	783		458	325	
	Vigorous activity	292	147		88	59	

^*∗*^Chi-square.

**Table 4:** Demonstrates the crude and adjusted sociodemographic independent variables for the development of hypertension.

Sociodemographic factors	Crude analysis			Adjusted analysis		
	OR	CI	*p* value	OR	CI	*p* value
Male	1.521	1.332–1.737	**0.0001**	1.83	1.544–2.168	**0.0001**
Female^*∗*^	Reference			Reference		

High educational level	0.218	0.181–0.264	**0.0001**	0.935	0.731–1.195	0.592
Medium educational level	0.369	0.307–0.445	**0.0001**	0.923	0.741–1.149	0.471
Low educational level^*∗*^	Reference			Reference		

No-alcohol use	1.247	1.093–1.423	**0.001**	1.018	0.866–1.197	0.827
Alcohol use^*∗*^	Reference			Reference		

Nonsmoking	1.285	1.097–1.505	**0.002**	0.981	0.815–1.181	0.839
Smoking^*∗*^	Reference			Reference		

Vigorous activity	1.433	1.065–1.929	**0.018**	1.492	1.057–2.104	**0.023**
Moderate activity	1.41	1.111–1.789	**0.005**	1.341	1.023–1.758	**0.034**
Walk	1.264	0.965–1.655	0.09	1.143	0.842–1.553	0.391
No activity^*∗*^	Reference			Reference		

Gozo	1.727	1.329–2.245	**0.0001**	1.771	1.303–2.408	**0.0001**
Northern	1.032	0.817–1.304	0.794	1.124	0.857–1.474	0.399
Western	1.424	1.125–1.802	**0.003**	1.473	1.121–1.937	**0.005**
Southeastern	1.541	1.229–1.9332	**0.0001**	1.674	1.287–2.178	**0.0001**
Northern Harbour	1.241	1.02–1.51	**0.031**	1.204	0.961–1.509	0.107
Southern Harbour^*∗*^	Reference			Reference		

Not in employment	2.421	2.114–2.772	**0.0001**	1.181	0.974–1.432	0.091
Employed^*∗*^	Reference			Reference		

BMI	1.127	1.113–1.141	**0.0001**	1.104	1.088–1.210	**0.0001**

Age	1.07	1.07–1.08	**0.0001**	1.066	1.058–1.073	**0.0001**

^*∗*^Reference category.
